# A One-Sided Affair: Unoriginal Origin of the Left Coronary Artery, a Case Report

**DOI:** 10.14740/cr331w

**Published:** 2014-10-06

**Authors:** Omair Ali, Saifur Rehman, Yaser Jbara, Bryan White

**Affiliations:** a Department of Internal Medicine, Boonshoft School of Medicine, Wright State University, Weber CHE Building, Second Floor, 128 East Apple Street, Dayton, OH 45409-2902, USA; b Department of Neurology and Psychiatry, Saint Louis University, 1438 S Grand Blvd, St. Louis, MO 63104, USA; c Department of Cardiology, Boonshoft School of Medicine, Wright State University, Weber CHE Building, Second Floor, 128 East Apple Street, Dayton, OH 45409-2902, USA

**Keywords:** Congenital coronary anomaly, Chest pain, Angina

## Abstract

Coronary artery anomalies constitute a group of congenital malformations that have a multitude of clinical manifestations and highly variable pathophysiology. We report a 56-year-old male with angina due to an anomalous origin of the left main coronary artery; approach and management.

## Introduction

We report a 56-year-old gentleman found to have an anomalous origin of the left main coronary artery.

## Case Report

A 56-year-old gentleman with a history of coronary artery disease of undefined anatomy or intervention in the past, presented to our cardiology clinic for evaluation of new onset angina. He described a substernal chest pressure occurring occasionally on exertion, intermittent, non-radiating, progressively getting worse, not associated with other symptoms. He endorsed a history of similar presentation and underwent percutaneous coronary intervention with stent placement.

### Investigations

**Figure 1:**
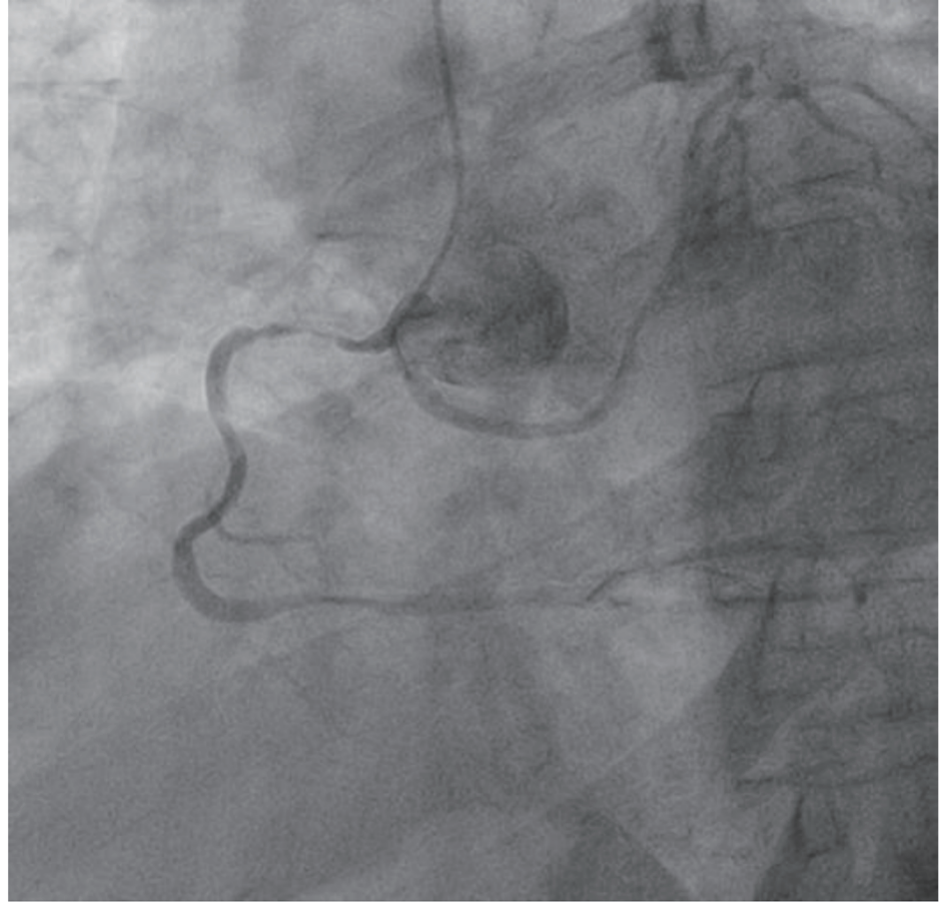
Coronary angiogram showing origin of the left coronary artery (LCA) and right coronary artery (RCA) from the right coronary cusp.

**Figure 2:**
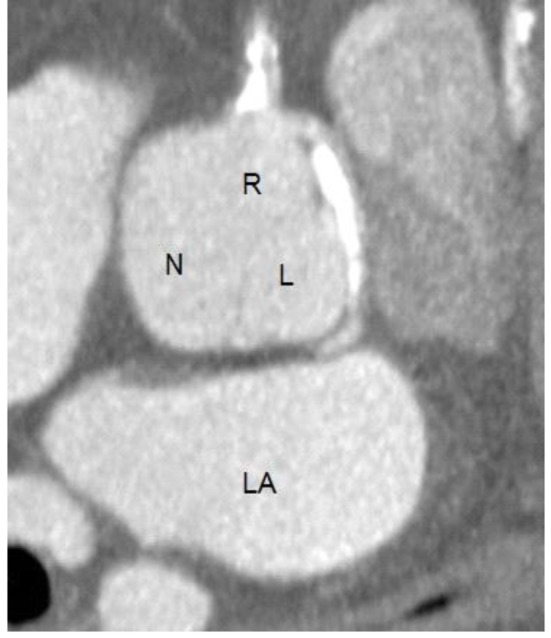
Coronary CT angiogram showing the right (R), left (L) and non-coronary (N) cusps. The right coronary cusp bares the ostia of the right coronary artery as well as the left coronary artery. The left atrium (LA) is also visualized.

**Figure 3:**
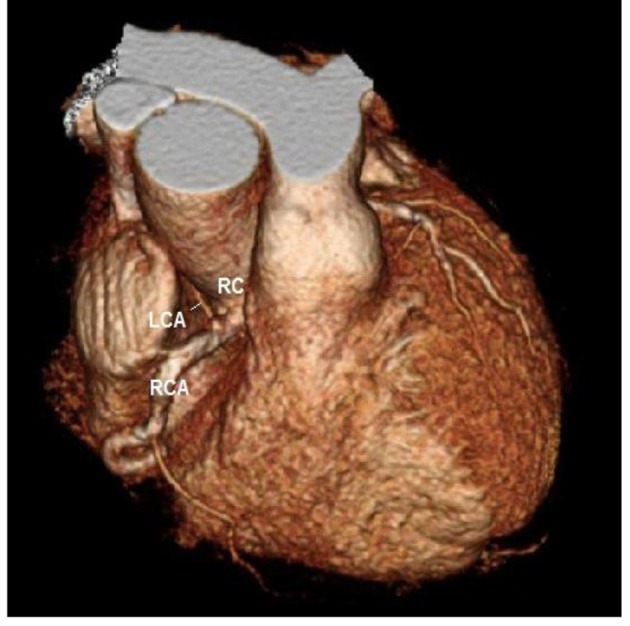
Coronary CT angiogram showing the RCA and LCA originating from the right coronary cusp.

**Figure 4:**
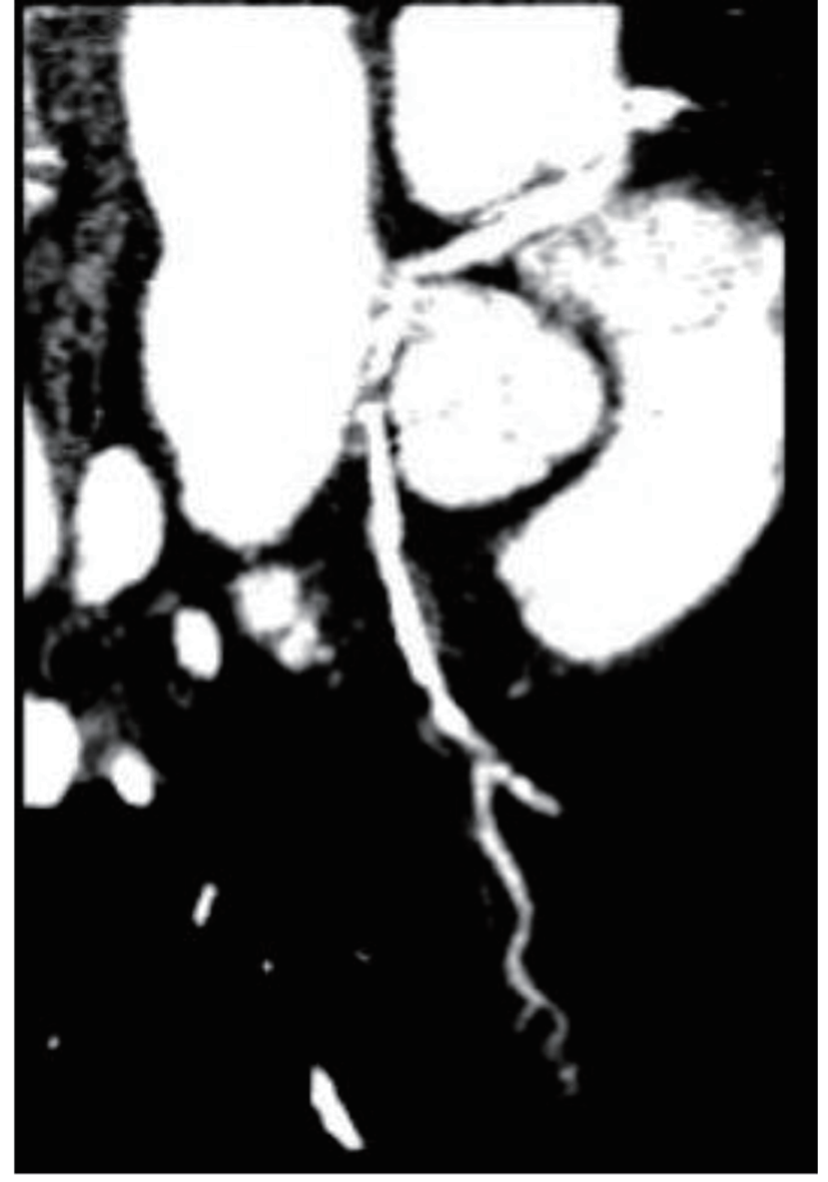
Coronary CT angiogram highlighting the origin of two vessels, the RCA and LCA, from a single coronary cusp.

He was referred for a Cardiolite stress study which showed a fixed inferior defect with ischemia. On coronary angiogram (Fig. 1), the left main coronary artery was found to have an anomalous origin of the right coronary cusp (from the aorta). It was found to have a 75% distal lesion. A 99% stent restenosis was also revealed. The rest of the angiogram had patent flow and the ventriculogram showed a ventricular function of 75%. A coronary CT angiogram (Fig. 2-4) also revealed this peculiar origin of the left main artery from the posterior margin of the right coronary cusp, extending posterior to the aortic root, between the aorta and left ventricle.

### Outcome

He was referred for a coronary bypass in which he got a left internal mammary artery (LIMA) to diagonal (artery), saphenous vein graft (SVG) to marginal (obtuse) and right coronary artery. He did well in recovery, meeting his goals and was discharged to follow-up.

## Discussion

Coronary artery anomalies constitute a group of congenital malformations that have a multitude of clinical manifestations and highly variable pathophysiology. The normal anatomy of the coronary arteries is characterized by two ostia centrally placed in the left and right aspects of the sinus of Valsalva just above the left and right coronary cusps respectively. The main left coronary artery (LCA) originates from the left ostium and branches into the left anterior descending artery (LAD) and the circumflex artery (LCX) which travels around the left atrioventricular groove. The right coronary artery (RCA) arises from the right ostium providing an infundibular branch to the anterior side of the heart; then courses back up in the atrioventricular groove. The main coronary arteries branch superiorly to the atria and inferiorly to the ventricles; they end in short fanning branches that penetrate the myocardium [1]. In order to address the normal spectrum of variations in this architecture however, it has been suggested that any form prevalent in > 1% of the general population be considered normal [2, 3].

Hemodynamically significant coronary artery anomalies may be classified as primary or secondary. The primary congenital coronary artery anomalies include anomalous origin of the coronary arteries from the aortic sinus, anomalous origin from the pulmonary artery, coronary artery stenosis, absent coronary artery, or coronary artery fistula. The secondary forms occur in conjunction with congenital heart disease [1].

In a necropsy study reported by Alexander and Griffith, the incidence of primary coronary anomalies in a series of 18,950 patients was 0.3% [4]. More recent studies however, suggest a much higher figure of 5.6% in patients studied by coronary angiography [2]. The large difference may be a result of strict diagnostic criteria and warrants further study into the subject.

These rare anomalies are usually detected with abnormalities in myocardial perfusion or hemodynamic abnormalities or in cases with progressive atherosclerosis. They can result in mild symptoms such as dyspnea to having severe manifestations such as sudden death. Cases with milder symptoms are usually not detected during life and even on post-mortem examination [1].

Anomalous origin of the LCA is a very rare clinical entity. It may arise from a number of sites, most importantly the pulmonary artery and right sinus of Valsalva (RSOV). Ectopic origin of the coronary artery from the opposite sinus of Valsalva may have important clinical manifestations such as exertional sudden cardiac death especially in younger patients [5]. In a study of 1,950 patients undergoing coronary angiography, only 0.15% were reported to have anomalous origin of the LCA from the RSOV [2]. Sudden cardiac death has particularly been notified among young adults with this anomaly [6].

The clinical syndrome depends on the route of the aberrant left main; it may have an intramural course between the aorta and pulmonary artery, an intraseptal course, a wraparound course in the posteroanterior interventricular groove, or it may run anterior to the pulmonary outflow [7, 8]. Compression of the LCA can occur because of the anterior course where it runs between the pulmonary artery and the aorta. This can result in poor perfusion of the myocardium leading to ischemia manifested by chest pain or even sudden death [9]. It has not yet been established whether those patients in whom the left main courses within the septal muscles as opposed to taking an interarterial route, are at a lower risk of sudden cardiac death [6].

Coronary angiography remains the gold standard for the diagnosis of coronary anomalies [1] as opposed to echocardiography which may give false negatives [10]. However, echocardiography, computed tomography and magnetic resonance imaging have been recommended as screening entities [11, 12]. Intravascular ultrasound may be used to assess the extent of stenosis and the need for interventional treatment [13, 14].

There are three options to treat symptomatic patients with such anomalies. Medical treatment (B-blockers) may be as effective as lifestyle modifications (avoidance of strenuous activity) in such patients [15]. Percutaneous coronary intervention with stent placement may be a reasonable option for anomalies with interarterial coursing and risk of systolic compression [13]. Early surgical correction has been the mainstay of treatment for many years and has shown the best outcome [16]. For anomalous origin of the LCA from the right cusp, surgical intervention has been highly recommended and it may be in the form of coronary artery bypass grafting to re-implant the ectopic artery into the left sinus of Valsalva, or by creating a longitudinal opening in the wall of the aorta in the intramural segment of the anomalous artery (osteoplasty) [8, 14].
